# Editorial: Omics Technologies Toward Systems Biology

**DOI:** 10.3389/fgene.2021.756847

**Published:** 2021-09-14

**Authors:** Fatemeh Maghuly, Gorji Marzban

**Affiliations:** Department of Biotechnology, BOKU-VIBT, University of Natural Resources and Life Sciences Vienna, Vienna, Austria

**Keywords:** multi-omics, data integration, functional genomics, intrinsic and extrinsic stressors, living organisms

By the end of Twenty century, analytical methodologies were enabled to explore thousands of biomolecules obtained from any given organisms. Moreover, numerous rising techniques qualified our laboratories to produce enormous amounts of data at different levels by several omics’ approaches with exceptional precision, resulting in development of databases and resources (Kumar et al.). However, the dilemma of obtained data remains persistently elaborating information due to the high number and volume. Besides, identification of biomolecules without a comprehensive understanding of the complexity underlying the cellular mechanisms would not deliver much information. Therefore, systems biology employs multi-omics platforms and computational approaches for data integration in different contexts (Krassowski et al.).

Nevertheless, analysis of different types of biomolecules requires extraction protocols compatible with the analytical instrumentation. Therefore, to conduct multi-omics efficiency, aliquots of the same sample are required for different extraction procedures optimized for different biomolecules, thereby decreasing sample handling time and increasing throughput. Kang et al. adapted a biphasic fractionation to extract proteins, metabolites, and lipids from one single sample (3-in-1) for liquid chromatography-tandem mass spectrometry (LC-MS/MS). The results showed that their method has great value to multi-omics and systems biology toward understanding the cellular networks, traits and phenotypes.

On the other hand, the combination of multi-omics data provide useful insight into the flow of biological information at multiple levels, thus can help to elucidate the complex mechanisms controlling the biological condition. For example, Guo et al. combined transcriptome and metabolome data obtained from clonally propagated plants at four developmental stages and three different environments to identify the spatial-temporal variation of flavonoids biosynthesis in leaves of Ginkgo. They indicated that flavonoids content varied considerably at different developmental stages and environments. Therefore, they expect that the accurate selection of planting region(s) and optimization harvesting time would substantially improve the production and management of Gingko in an industrial manner.

In the frame of cell factories and selected targets, multi-omics and systems biology reflect a challenging area for the engineering of cellular metabolism and maximizing the production of valuable compounds through bioconversion. *In silico* experiments were shown to replace time and laborious processes to win information about the cell networks. Tafur Rangel et al. proposed that computational tools and metabolic modeling in combination with transcriptomics can accelerate the optimization of cell factories by identifying key metabolic engineering targets (genes/reactions) and not only by predicting mutants. However, It depends on the level of completeness and accuracy of the metabolic model, which could be improved by omics data.

Dictated by the rapidly growing worldwide human population, increased agricultural productivity is necessary to cope with the food demand. In this context, multi-omics technologies have helped plant biologists complete their understanding of plant metabolism by reconsidering and identifying novel pathways. Kumar et al. represented how this new knowledge can be utilized to develop improved cultivars by targeting metabolic pathways and use this information for re-domestication and *de novo* domestication of wild relatives.

Considering that combination of two or more omics data sets in data analysis, visualization and interpretation are essential to determine the mechanism of a biological process; Krassowski et al. provided an excellent overview of the current state of the field, inform on available reliable resources, discuss findable, accessible, interoperable, reusable research, and point to best practices in benchmarking. They also addressed challenges with biological complexity, acknowledged current tools limitations, and concluded future perspectives in this field.

Accordingly, systems biology tools made it possible to develop personalized medicine directly related to analyzing huge amounts of data delivered by high throughput technologies. Pires et al. described how to perform the translation from RNA-seq data into therapeutic targets. They present an online platform using the MEAN stack supported by a Galaxy pipeline for translating RNA-seq data into protein targets suitable for the chemotherapy of solid tumors.

To gain new insight into the evolution of extremophiles and the actual limits for life, in-depth knowledge of proteome-related alterations in cell physiology is crucial. Furthermore, in extreme environments, microbial extremophiles are of great interest to understand stress adaptation and survival mechanisms. Therefore, Tesei et al. pioneered the qualitative and quantitative proteomic analyses on the mycelia, a lack fungi, and supernatant of culture medium to show its ability to cope with microgravity, which has significance to exobiology and implications to planetary protection policies.

Given that acidification of arable lands is one of the biggest problems of modern agronomy, Szurman-Zubrzycka et al. studied the global transcriptome of root meristematic cells from barley grown at low pH treated with aluminium. They showed that low pH is a stress factor; however, aluminium causes more changes at the transcriptome level by long term stress. Thus, aluminium toxicity in acidic soils, resulting in inhibition of both elongation and division rates of root cells, consequently reducing water and nutrient uptake and finally reducing growth and yield.

Taking together, living organisms are innately exposed to a wide range of intrinsic and extrinsic sources, causing damage to the DNA, thereby promoting genomic instability. To escape the harmful effects, organisms harbor several DNA damage repair (DDR) pathways. Because plants are sessile, they have involved highly conserved DDR pathways that share several components with other organisms. In this manner, they maintain their genetic integrity and transfer their accurate genetic information to subsequent plant generations. Raina et al. summarized these complex mechanisms by which plants repair their DNA from severe exposure to both biotic and abiotic stresses and how lack of the DDR pathway affects various developmental stages.

In addition, the sequencing of several genomes based on comparative approaches and recent discoveries of Small Open Reading Frames (small ORFs/sORFs/smORFs) peptides has been recently described as essential players in biological processes and opened new avenues for smORF research, reported as potential non-functional or junk DNA. In this context, Guerra-Almeida and Nunes-da-Fonseca represent intriguing questions to debate further investigation and future perspectives for the non-functional smORF peptides.

On the other hand, an effective high throughput functional genomics tool for studying genes responsible for desired phenotypes is required to facilitate genome-wide investigations. TILLING (Targeting Induced Local Lesions IN Genomes) is a powerful reverse genetics method in plant functional genomics; however, one of the main challenges for a successful TILLING experiment is that currently available bioinformatic tools for variant detection are not designed to identify mutations with low frequencies or to perform sample identification from variants in overlapping pools. To overcome this shortage, Gil et al. developed, through the Next Generation Sequencing Experience Platform, two novel functionalities for TILLING: a TILLING experiment simulator and a TILLING detector. These new bioinformatic tools increase the precision of TILLING experiments, which is useful for implementing TILLING as a tool for functional genomics and breeding.

In this context, Guo et al. also identified loss-of-function mutations in blast susceptible genes through TILLING by sequencing. Furthermore, they suggested that identified mutants might also provide enhanced immunity with severe effects on protein function and resistance to wheat blast. Thus, the study provides a new strategy, novel resistant lines, and valuable gene resources to tackle disease-resistant wheat breeding.

We wish to thank all contributors to this special issue and hope that its appearance provides interest to users recent and novel research trends in the application of omics technologies.

